# Focus on Interferon Signature in Cutaneous Lupus Erythematosus: Novel Therapies From Better Understanding of the Pathogenesis

**DOI:** 10.1155/jimr/5600731

**Published:** 2025-10-14

**Authors:** Min Gao, Nenghan Zhang, Yumin Xia

**Affiliations:** ^1^ Department of Dermatology, The Second Affiliated Hospital of Xi’an Jiaotong University, Xi’an, 710004, China, xjtu.edu.cn

**Keywords:** cutaneous lupus erythematosus, cytokine, inflammation, interferon, targeted therapy

## Abstract

Cutaneous lupus erythematosus (CLE), a common clinical manifestation of lupus erythematosus (LE), significantly compromises patients’ quality of life and social functioning due to its relatively high prevalence. While the exact pathogenesis of CLE remains incompletely understood, accumulating evidence highlights the pivotal role of interferon (IFN) as a central mediator in disease initiation and progression. Stromal cells and infiltrating immune cells within CLE lesions demonstrate elevated expression of IFN‐stimulated genes (ISGs), establishing a characteristic IFN signature. IFN orchestrates multiple pathological processes, including chemokine‐mediated immune cell recruitment, cutaneous inflammation cascade, and tissue fibrosis. This review systematically examines the IFN‐CLE axis through an integrated analysis of in vitro and in vivo experimental data. Emerging clinical trials reveal therapeutic promise in strategies targeting plasmacytoid dendritic cells (pDCs), neutralizing IFN signaling, or blocking downstream pathways. Given the current limitations in CLE management, IFN‐focused strategies may offer innovative solutions to address unmet clinical needs through precision immunomodulation.

## 1. Introduction

Lupus erythematosus (LE) encompasses a spectrum of autoimmune diseases, with cutaneous LE (CLE) and systemic LE (SLE) representing distinct clinical entities. CLE may present as an isolated dermatologic disease or as a cutaneous manifestation of SLE, comprising four primary subtypes: acute, subacute (SCLE), chronic, and intermittent forms. Discoid LE (DLE) constitutes the most prevalent chronic subtype [1, 2]. With an estimated incidence of 4.0–4.3/100,000 individuals, approximately 5%–25% of CLE patients progress to SLE, while 70%–85% of SLE patients develop skin involvement [3–7], significantly impairing their quality of life and social functioning. The disease exhibits marked sexual dimorphism, showing 9:1 female predominance in reproductive‐aged populations compared to equal gender distribution in pediatric cases [8], potentially linked to estrogen influence.

The pathogenesis of CLE involves multifactorial contributors, including genetic predisposition, environmental triggers, infections, and medications [1]. Ultraviolet radiation (UVR) is a predominant environmental inducer, reducing cutaneous tolerance thresholds and amplifying photosensitivity. This phototoxic effect drives the upregulation of interferon (IFN) and IFN‐stimulated genes (ISGs) across keratinocytes, plasmacytoid dendritic cells (pDCs), and other cutaneous cell populations, initiating pathological cascades [9]. IFNs comprise three classes of cytokines differentiated by receptor specificity and signaling mechanisms.

IFNs bind to their respective receptors to activate downstream signaling pathways, primarily the Janus kinases (JAK)‐signal transducer and activator of transcription (STAT) pathway. The type I IFN family includes 17 members, including IFN‐α1 to IFN‐α13, IFN‐β, and IFN‐κ, which bind to the IFN‐α receptor (IFNAR) to exert antiviral and immunomodulatory effects [10–13]. The type II IFN consists mainly of IFN‐γ, produced by activated T cells and natural killer (NK) cells, which enhances macrophage activity, promotes T cell differentiation, and modulates immune responses [5]. The type III IFNs bind to their receptor and play a role in pathogen barrier defense against pathogens. Due to the restricted expression of IFN‐λ receptor (IFNLR), IFN‐λ primarily acts on epithelial cells [14–16]. This intricate interplay of IFN hyperactivation and receptor‐specific signaling cascades underscores the molecular foundation of cutaneous inflammation.

Recently, a growing body of research has highlighted the role of the IFN signature in LE pathophysiology, referring to the significant upregulation of specific genes following IFN stimulation. This review summarizes the pathogenic role of IFN in CLE and focuses on the translational progress of targeting IFN‐related cells and pathways for the therapeutic intervention.

## 2. The Increased Expression of ISGs in CLE Lesions

Differential gene expression analyses of CLE lesions reveal upregulated genes involved in innate and adaptive immune activation, including nucleic acid recognition mechanisms (e.g., Toll‐like receptor [TLR] signaling and cytoplasmic DNA sensing) and downstream pathways [17]. Lesion samples from SCLE and chronic CLE exhibited similar expression profiles, with high expression of ISGs (activated by IFN, particularly type I IFN) indicating enhanced IFN activity [18, 19]. In DLE lesions, fibroblasts demonstrate a strong IFN signature alongside transforming growth factor (TGF)‐β‐induced collagen response [20]. Thus, CLE lesions are characterized by upregulated innate/adaptive immunity genes, shared ISG/IFN signatures, and fibroblast‐specific IFN/TGF‐β responses.

**Figure 1:**
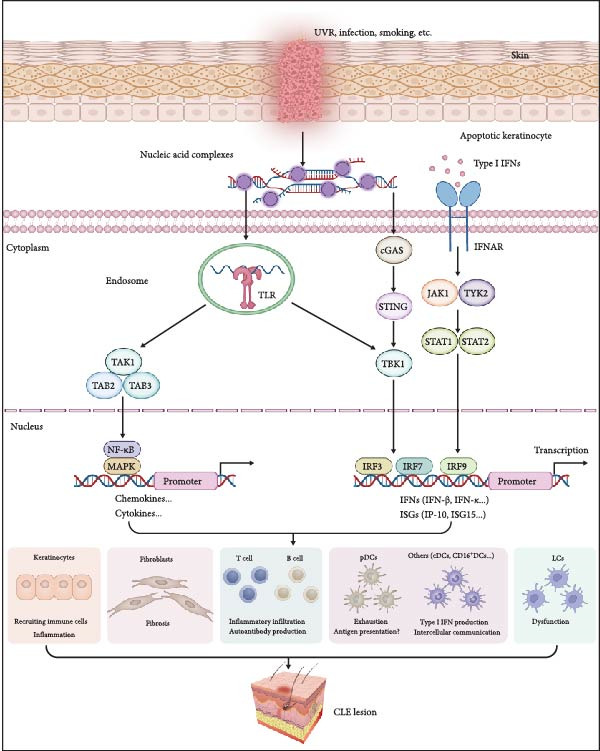
The pathogenic role of interferons in cutaneous lupus erythematosus. UVR, infection, and other stressors induce apoptosis in keratinocytes, releasing nucleic acid complexes. These complexes activate immune cells via TLR and STING pathways, triggering type I IFN production. Type I IFNs and other cytokines promote inflammation, fibrosis, and immune cell recruitment, escalating the disease. Key cellular players include keratinocytes, fibroblasts, T/B cells, pDCs, and CD16 ^+^ DCs, etc., which together drive the inflammatory cascade and tissue damage characteristic of CLE lesions. IP‐10, interferon‐induced protein 10; IRF, interferon regulatory factor; MAPK, mitogen‐activated protein kinases; NF‐κB, nuclear factor kappa‐light‐chain‐enhancer of activated B cells; TAB, TAK1 binding protein; TAK1, TGF‐β activated kinase 1; TBK1, TANK binding kinase 1. The “?” means the antigen presentation.

As shown in Figure 1, UVR synergizes with factors, such as infection and smoking to contribute to CLE pathogenesis, serving as a significant trigger. In both humans and mice, UVR exposure increases ISGs levels in the skin and peripheral blood, with a more pronounced effect observed in female mice [21, 22]. A comparison of UVR‐induced genes in healthy, DLE, and SCLE skin identified 411 differentially expressed genes, with ISGs specifically upregulated in DLE and SCLE lesions [9]. DLE lesions, unlike other CLE types, exhibit gene expression profiles that promote fibrosis and scarring. In vitro studies of DLE samples demonstrate time‐dependent expression of fibrotic markers [23]. Obviously, UVR triggers CLE via ISGs upregulation in lesions and DLE‐specific fibrotic responses.

### 2.1. Skin Stromal Cells Exhibit a Distinct IFN Signature in CLE Lesions

Keratinocytes and fibroblasts in skin stroma display significant and pronounced IFN signatures. Keratinocytes highly express ISGs even in non‐lesional skin, enhancing antiviral capability and immune responses and promoting chemokine secretion to attract immune cells [24]. Primary keratinocytes derived from LE patient lesions and immortalized human keratinocytes overexpressing IFNK both exhibit a robust IFN signature, which is maintained by IFN‐*κ* expression [25]. In contrast, this IFN signature is abolished in IFNK knockout immortalized human keratinocytes [25].

Fibroblasts with a strong IFN signature in CLE lesions are primarily concentrated in the superficial dermis, where they interact with IFN secreted by keratinocytes, promoting skin inflammation and fibrosis [1, 24]. The addition of conditioned medium from keratinocytes to fibroblast cultures significantly upregulates the expression of IFN‐responsive genes (e.g., IRF7) in fibroblasts [25]. This upregulation can be blocked by an IFN‐*κ* neutralizing antibody, whereas neutralizing antibodies against other IFNs (e.g., IFN‐α and IFN‐β) have no such effect, indicating that keratinocyte‐derived IFN‐*κ* influences adjacent stromal cells via paracrine signaling [25]. These findings suggest that cutaneous keratinocytes and fibroblasts exhibit IFN‐driven ISGs, enhancing antiviral immunity and fibrosis via chemokine secretion.

#### 2.1.1. Keratinocytes Serve as Both Producers and Responders of IFN in CLE

Research indicates that the effects of UVR and IFNs on keratinocytes are associated with lesions in lupus patients. In vitro, synthetic ligands for pattern recognition receptors can induce high levels of multiple IFN subtypes in keratinocytes [17, 26, 27]. Although keratinocytes do not produce IFN‐α themselves, exogenous IFN‐α significantly enhances their susceptibility to UVR‐induced apoptosis, to the extent that even suberythemal UV doses can trigger cell death [9, 22, 28, 29]. This in vitro observation correlates with the clinically observed lower minimal erythema dose in SLE patients, a phenomenon primarily attributed to endogenous IFN overproduction.

A single dose of UVB exposure can induce a robust local type I IFN response via the cyclic GMP–AMP synthase‐stimulator of interferon genes (cGAS–STING) pathway in the skin. Subsequently, the type I IFN signal disseminates through the bloodstream, creating a systemic IFN signature and recruiting inflammatory cells, thereby directly linking cutaneous photosensitivity to acute SLE exacerbations [22]. UVB radiation damages keratinocyte mitochondria, leading to the release of mitochondrial *Z*‐DNA. The *Z*‐DNA‐binding protein 1 (ZBP1) stabilizes this DNA and enhances type I IFN production via the cGAS–STING pathway. Furthermore, ZBP1 accumulates in photosensitive skin areas of lupus patients, further supporting the role of IFNs in the pathogenesis [30]. Hence, UVB‐triggered mitochondrial DNA release and ZBP1‐mediated cGAS–STING activation drive systemic IFN responses, directly connecting photosensitivity to lupus flares.

IFN‐*κ*, a type I IFN, is markedly elevated in both lesional and non‐lesional skin of CLE patients and is specifically localized to the epidermis [25]. In *IFNK* knockout keratinocytes, baseline phosphorylated‐STAT1/2 levels are reduced after UVR exposure, with a diminished IFN‐α response and minimal apoptosis. Conversely, mouse epidermis overexpressing *IFNK* shows an enhanced IFN‐α response and increased apoptosis post‐UVR [25, 28]. Thus, IFN‐*κ* amplifies IFN‐α response and UVR‐induced apoptosis in CLE keratinocytes and epidermis.

IFN‐*κ* expression depends on the IFN‐β signaling pathway. In human keratinocytes, IFN‐β expression precedes IFN‐*κ* expression following stimulation with nucleic acid analogs or UVR. Neutralizing IFN‐β inhibits IFN‐*κ* upregulation, and *IFNB1* knockout abolishes *IFNK* expression [11]. Interleukin (IL)‐6, a pathogenic proinflammatory cytokine in CLE, promotes T cell differentiation and B cell maturation. Treatment with IFN‐*κ* increases IL‐6 production in normal keratinocytes, while blocking IFN‐*κ* reduces IL‐6 secretion in lupus keratinocytes [31, 32]. However, targeting IL‐6 and its receptor (e.g., with Sirukumab) has shown no therapeutic efficacy in CLE [33, 34]. Nonetheless, IFN‐*κ* expression relies on IFN‐β signaling and drives IL‐6 production in CLE.

IFN‐λ and its receptor are elevated in CLE lesions and serum. By activating keratinocytes, IFN‐λ upregulates ISGs and proinflammatory factors, driving skin inflammation. However, in Imiquimod‐treated *Ifnlr1* ^−/−^ mice, skin inflammation and proinflammatory cytokine expression are reduced [35, 36]. When IFN‐λ and IFN‐α act synergistically on keratinocytes, they induce higher chemokines expression, reflecting the in vivo environment where keratinocytes are exposed to multiple cytokines [35]. Thus, IFN‐λ synergizes with IFN‐α to exacerbate skin inflammation via ISGs and cytokines.

#### 2.1.2. Fibroblasts May Play a Significant Role in the Fibrosis of DLE

Fibroblasts proliferate significantly in DLE and SCLE lesions, with specific subpopulations exhibiting a strong IFN signature [1, 20]. Upon stimulation with key CLE cytokines, such as IFN‐γ and IFN‐α, fibroblasts upregulate CXCL chemokines and ISGs, promoting immune cell recruitment and activation [37, 38]. Primary fibroblasts from non‐lesional LE skin also show higher inflammatory gene expression upon stimulation with IFN‐γ and IFN‐α [20]. Additionally, fibroblasts express IFNLR, which is upregulated by IFN‐α. IFN‐λ1 enhances type I, IV, and VII collagen expression induced by TGF‐β1 but not type III collagen [14], potentially contributing to scarring in CLE lesions.

### 2.2. The IFN Signature in Immune Cells of CLE

In CLE, the IFN signature is prevalent across various immune cells. T cells with high ISGs expression are found in both skin lesions and peripheral blood of CLE patients [24, 38, 39]. Research reveals that in CLE lesions, epidermal T cells mainly respond to type I IFN signaling, while dermal T cells are involved in viral responses and IFN‐γ modulation [38]. Similarly, a B cell subpopulation expresses high levels of ISGs and heat shock protein genes, indicating type I IFN activation and potential involvement in antigen presentation [38]. Hence, both T and B cells indicate type I IFN activation in CLE lesion.

#### 2.2.1. A Variety of T Cells Involved in CLE Lesions

CLE is primarily characterized by type I IFN‐amplified T helper (Th) 1 cell‐mediated inflammation. Transcriptomic profiling of DLE lesions shows an IFN‐λ‐dominated Th1 signature [40]. IFN‐α stimulates keratinocytes to secrete CXCL9 and CXCL10, which recruit CXCR3^+^ T cells into the skin [41–44]; locally recruited Th2 cells are subsequently converted to a Th1 phenotype under the influence of type I IFN [45].

Apoptotic keratinocytes release endogenous nucleic acids (eNAs) into the dermal milieu [46]. Within CLE lesions, these eNAs engage endosomal TLR7/9 and cytosolic sensors to trigger a robust type I IFN response that transcriptionally upregulates CXCL9 and CXCL10 [9, 17, 26]. The resultant chemokine gradient orchestrates the selective recruitment of CXCR3^+^ T cells from the peripheral circulation into lesional tissue [41–43]. Consequently, CXCR3‐expressing T cells account for 60%–90% of lesional infiltrate [42], while their circulating counterparts are proportionally reduced [41].

Concomitantly, lesional IFN‐α induces quantitative reduction and functional impairment of regulatory T cells (Tregs) by downregulating forkhead box protein P3 (FoxP3) and IL‐2 [24, 26, 44, 47]. Clinically, low‐dose IL‐2 therapy selectively expands the Treg compartment and improves CLE lesions [48]. Additionally, anti‐CD19 CAR‐Treg engineered to overexpress FoxP3 effectively restrains B cell activation in vitro and restores immune homeostasis [49]. Thus, IFN‐α‐mediated Treg dysfunction drives lupus pathogenesis, while IL‐2 therapy and engineered Tregs offer promising immunomodulatory strategies.

CLE skin harbors an expanded pool of memory T cells, including CD4^+^ and CD8^+^ tissue‐resident memory T (Trm) cells. SCLE and DLE lesions contain more CD4^+^ Trm cells compared to acute CLE, suggesting a more critical role in persistent lesions [39, 50, 51]. Additionally, compared to healthy individuals, peripheral helper T cells or follicular helper T cells in LE skin lesions show a strong IFN signature and high expression of CXCL13 and costimulatory molecules, which may enhance local inflammation and B cell activation [24, 39]. This indicated that CLE patients exhibit elevated CD4^+^ Trm cells in skin lesions, linked to persistent inflammation and B cell activation.

#### 2.2.2. The Infiltration of B Cells in CLE Lesions

B cell infiltration in CLE is heterogeneous. Some chronic CLE patients have B cells similar to healthy individuals, while others show SLE‐like B cell abnormalities, such as effector B cell proliferation and autoantibody production, albeit generally at lower autoantibody levels [52, 53]. DLE patients, especially those without SLE, have more prominent B cell gene signatures with higher immunoglobulin gene expression [54, 55].

B cell activating factor (BAFF) expression is elevated in CLE, and in vitro IFN‐α stimulation of keratinocytes increases BAFF and its receptor mRNA levels [56, 57]. In SLE, B cells from patient sera respond to IFN‐λ by upregulating IFNLR1 and ISGs [35]. IFN‐λ acts synergistically with type I IFNs to enhance B cell responsiveness to IFN stimulation [58], although it neither activated B cells nor influenced autoantibody titers in the imiquimod‐induced mouse lupus model [35]. This discrepancy suggests that IFN‐λ’s impact on B cells may be governed not by intrinsic ligand activity but by two context‐dependent variables: (i) the species‐specific density of IFNLR1 on B cells and (ii) the local concentration of IFN‐λ.

#### 2.2.3. Non‐pDC Myeloid Cells Might Drive Type I IFN Production in LE Lesions

pDCs represent the most numerous dendritic cells (DCs) population in non‐lesional skin of CLE patients [24]. Upon TLR9 stimulation, only a minor fraction of pDCs from healthy donors secrete IFN‐α, and no discrete pDC subset predicts type‐I IFN output under homeostatic conditions, implying a stochastic activation process [59]. Within the tissue microenvironment, IFN‐α can trigger adjacent pDCs to release additional IFN‐α, and the magnitude of secretion rises with local pDCs density, indicating that paracrine signaling and cell density jointly govern pDC activation [59]. As noted earlier, UVR induces keratinocyte apoptosis and the formation of nucleic acid‐containing immune complexes, which bind low‐affinity pDC receptors, are internalized, and drive type I IFN production [60–62].

Historically, pDCs were viewed as the principal source of type I IFN in human and murine blood and lymphatic tissue. However, recent single‐cell analyses challenge this paradigm. Within established CLE lesions, DCs and monocyte/macrophage subsets—not pDCs—produce more type I IFNs [63, 64]. In CLE patient skin sections, mRNA in situ hybridization revealed no colocalization of IFN‐α1 or IFN‐β transcripts with pDC markers [63]. Moreover, pDC function in lupus patients may be exhausted. The impaired function of pDCs is manifested by a weakened response to TLR7 and TLR9 agonists, resulting in an inability to produce substantial amounts of IFN‐α and tumor necrosis factor [63, 64].

In addition, CD16^+^ DCs and monocyte/macrophage subsets within CLE lesions display heightened type I IFN pathway activity, suggesting that non‐pDC myeloid cells may compensate by upregulating IFN production [63]. Nonclassical monocyte‐derived CD16^+^ DCs are markedly expanded in both lesional and non‐lesional skin of CLE patients [24]. These CD16^+^ DCs sustain high type I IFN output, driving keratinocytes to amplify IFN‐*κ*, IL‐6, and other cytokines in a positive feedback loop that escalates inflammation and recruits immune cells [24]. Together with keratinocytes, these cells might constitute the primary source of type I IFNs.

#### 2.2.4. Dysfunction of Langerhans Cell (LC) Promotes the Progress of CLE

LCs are epidermal resident DCs that express langerin and coordinate immune responses against skin pathogens [65, 66]. Normal skin can suppress inflammation after UVR exposure through LC‐mediated phagocytosis of apoptotic cells [67]. However, abnormal distribution and reduced LC numbers in lupus skin may potentially cause loss of tolerance to skin antigens [24, 65]. Inducing LC depletion in adult lupus‐prone mice increases autoantibodies targeting skin antigens, significantly accelerating the onset of lupus‐like skin lesions and enhancing local macrophage infiltration [65].

UVR activates a disintegrin and metalloproteinase 17 (ADAM17) in LCs, boosting epidermal growth factor receptor (EGFR) ligand expression and activating EGFR, which limits UVR‐induced keratinocyte apoptosis and skin damage [68, 69]. Notably, IFN‐*κ* and IFN‐β in skin significantly reduce UVR‐induced ADAM17 proteolytic activity in LCs. Anti‐IFNAR therapy restores LC ADAM17 activity and alleviates UVR‐induced skin inflammation and barrier dysfunction [70]. Targeting IFN signaling pathways may restore LCs function and mitigate skin inflammation.

#### 2.2.5. The Interaction Between Other Immune Cells and IFN in CLE Remains Investigating

Neutrophil extracellular traps (NETs), composed of DNA, histones, and neutrophil granules containing elastase and myeloperoxidase, can promote skin damage and are involved in initiating and perpetuating autoimmune responses. In CLE, NET formation is linked to type I IFN production, and NET release can activate TLRs in pDCs, further increasing type I IFN levels [71]. LE patients with skin lesions had higher NET‐forming neutrophils, suggesting a stronger association with tissue damage and scarring [72].

The number of NK cells in the peripheral blood of SLE patients is reduced [73, 74], possibly due to increased IFN‐α levels causing higher apoptosis of activated NK cells [73]. NK cells are enriched in inflammatory lesions and can proliferate in CLE skin lesions, but their exact pathogenic role remains unclear [75]. Investigating NETs and NK cell dynamics could unveil novel therapeutic targets in CLE pathogenesis.

## 3. Translational Progress in the Therapies of CLE

Current treatments for CLE include smoking cessation, photoprotection, topical corticosteroids, and calcineurin inhibitors. Systemic therapies involve antimalarials like hydroxychloroquine as the first‐line agents and immunosuppressants or immunomodulators reserved for severe cases. While biologics are approved for SLE, unmet needs remain. Biologics targeting the IFN pathway for CLE are under investigation and hold promise for future therapies [76, 77].

As depicted in Table 1, we summarized the translational progress of targeting the IFN pathway, including agents acting on pDCs, IFN itself, and the downstream JAK‐STAT pathway. Their specific information is detailed in the following text. The primary indicators for assessing skin symptom improvement are the reduction in the Cutaneous Lupus Erythematosus Disease Area and Severity Index (CLASI) [100]and the CLASI50 response, defined as a ≥ 50% decrease in CLASI score from baseline, which is a key efficacy endpoint.

**Table 1:** Summary of the translational progress of targeting IFN pathway and relevant information.

Target		Generic name	Outcome	Adverse effects	State
pDCs	BDCA2	Litifilimab [78]	CLASI‐A score dose dependently reduced at Week 16	SAEs including herpes zoster and herpes keratitis occurred	A Phase III clinical trial for CLE is currently underway
		CBS004 [79]	Inhibited skin inflammation and fibrosis in mouse models	—	Preclinical stage
		SSGJ‐626	—	—	Phase I trial (CTR20244183) is underway
		BD2304	—	—	Phase I trial (NCT06625671) is underway
	ILT7	Daxdilimab [80, 81]	Had a positive trend in reducing disease activity	Safe and well tolerated	Clinical trials are ongoing

IFN	IFNAR1	Anifrolumab [82–84]	About 50% patients achieved CLASI50 response at Week 52	Upper respiratory tract infection and herpes zoster were common	Approved for treating SLE
	IFN‐α	Sifalimumab [85]	More patients achieved CLASI improvement at Week 52	Herpes zoster was common	—
		S95021 [86]	Reduced STAT1 phosphorylation	—	Phase I trial is preparing
		Rontalizumab [87]	Did not achieve any primary or secondary endpoints	Safe and well tolerated	Research has stopped
		AGS‐009 [88]	Significantly neutralized the IFN signature	Safe and well tolerated	—
		IFN‐α kinoid [89–91]	Neutralized 13 IFN‐α subtypes but not effective	Safe and well tolerated	Research is at a standstill
	IFN‐γ	AMG 811 [5]	Did not observe the efficacy	Safe and well tolerated	—

JAK‐STAT pathway	TYK2	Deucravacitinib [92]	Improved CLASI50 response and skin symptoms	High incidence of rash and acne	—
	JAK1/2	Baricitinib [93, 94]	Showed contradictory results of two clinical trials in treating SLE	Safe and well tolerated	Research has stopped
		Ruxolitinib [95–97]	Nearly resolved skin lesions in treating chilblain lupus	—	—
	JAK1	Upadacitinib [98]	Improved CLASI50 response and skin symptoms	Possibility of increased risk of infection	The Phase III trial (NCT05843643) for SLE
	JAK1/3	Tofacitinib [99]	Partially ameliorated rash	No sAEs	—

Abbreviation: NA, not available.

### 3.1. Targeting pDCs

Human pDCs selectively express the surface markers blood DC antigen (BDCA)−2 and BDCA‐4, along with the highly pDC‐restricted receptor immunoglobulin‐like transcript (ILT) 7 [80]. Besides, BDCA2 can also be expressed on inflammatory monocytes [101]. Both BDCA2 and ILT7 independently associate with the FcεRIγ adaptor, forming two distinct pDC‐restricted receptor complexes [102–104]. Targeting BDCA2 and ILT7 to transiently deplete pDCs has shown therapeutic potential for SLE in vitro experiments [61, 80, 105]. Some monoclonal antibodies targeting BDCA2 and ILT7 have advanced to clinical trials with promising results.

#### 3.1.1. Targeting BDCA2

Litifilimab (BIIB059) is a fully human monoclonal antibody targeting BDCA2. It reduces pDC numbers in a circulating Litifilimab level‐dependent manner [106]. SLE patients with IFN signature showed rapid neutralization of some ISGs within 24 h after a single 20 mg/kg intravenous dose. Four weeks postadministration, most patients experienced a lower CLASI score [106].

In the Phase II LILAC‐B trial, all Litifilimab dose groups (50, 150, and 450 mg) reduced CLASI score at Week 16 compared to vehicle [78]. Nevertheless, in the LILAC‐A trial involving SLE patients with joint and skin involvement, 56% of Litifilimab‐treated patients achieved a ≥ 7‐point reduction in CLASI score versus 34% in the vehicle group, though this difference was not statistically significant [107]. Regarding the safety, reported adverse effects (AEs) and severe AEs (SAEs) for Litifilimab were comparable to placebo; however, attention should be paid to varicella zoster virus reactiviaton [78, 107].

Overall, Litifilimab has shown favorable efficacy and safety in treating LE skin symptoms in clinical trials. However, due to limited sample size and short durations, longer and larger trials are needed to fully assess its profile.

#### 3.1.2. Targeting ILT7

Daxdilimab (VIB7734) is a humanized monoclonal antibody targeting ILT7. Its afucosylation enhances its cytotoxic effects on pDCs [80]. In two Phase I studies involving patients with autoimmune disease, Daxdilimab effectively depleted pDCs in both blood and skin [80]. In a Phase II trial for moderate‐to‐severe SLE, Daxdilimab significantly reduced blood pDCs after 48 weeks. Though the primary endpoint response rate showed no difference, positive trends in lupus activity scales suggest potential efficacy [108]. Daxdilimab is well‐tolerated and safe, with ongoing research exploring its use in other autoimmune diseases like DLE and dermatomyositis.

### 3.2. IFN Blockades

Given the prominent IFN signature in CLE, blocking the IFN pathway appears to be a potential therapeutic strategy. Currently, biologics primarily target IFNAR1, IFN‐α, and IFN‐γ.

#### 3.2.1. Targeting IFNAR1

Anifrolumab is a monoclonal humanized antibody targeting IFNAR1, effectively blocking all type I IFNs [7]. Having undergone Phase II and III clinical trials, it is approved by Food and Drug Administration and European Medicines Agency for treating moderate‐to‐severe active SLE [109], administered intravenously at a dose of 300 mg every 4 weeks.

Although not yet approved for the treatment of CLE, Anifrolumab has demonstrated significant potential in improving CLE skin symptoms. In Phase II/III trials [82–84], among patients with a CLASI score of ≥ 10 at baseline, 63% achieved a CLASI50 response at Week 52 with intravenous Anifrolumab 300 mg, compared to only 25%–30% in the vehicle group. Common AEs include upper respiratory infections and herpes zoster, with sAEs being rare in clinical trials.

Multiple case series [7, 109–111] have reported the use of intravenous Anifrolumab for the refractory CLE, including patients who had failed multiple systemic therapies. Anifrolumab has been shown to effectively manage rashes while reducing the need for oral medications like prednisone.

SLE patients treated with subcutaneous injections of 300 mg Anifrolumab demonstrated a trend of improvement in CLASI scores that increased with longer treatment duration. However, possibly due to the small sample size, the improvement in CLASI was not significant compared to the placebo group [112].

#### 3.2.2. Targeting IFN‐α

Sifalimumab is a fully human monoclonal antibody targeting IFN‐α. It competitively binds to IFN‐α2A and is used for SLE, dermatomyositis, and polymyositis [85, 113, 114]. Sifalimumab inhibits ISGs mRNA and protein levels in the skin in a dose dependent manner, correlating with rash improvement [115].

In a Phase II trial for moderate‐to‐severe active SLE, more patients in the Sifalimumab groups showed CLASI improvement at Week 52 compared to placebo, with statistical significance observed only in the 200 and 1200 mg groups but not in the 600 mg group. Safety‐wise, overall AEs and sAEs were similar between groups, but herpes zoster occurred more frequently with Sifalimumab, likely due to impaired antiviral defenses from IFN‐α blockade [85].

S95021, a fully human monoclonal antibody, binds to IFN‐α with higher affinity and broader neutralization than Sifalimumab. In vitro, S95021 dose dependently reduces STAT1 phosphorylation and IFN signature induced by IFN‐α or SLE patient plasma [86]. Its superior neutralization capacity suggests potential as a new treatment for autoimmune diseases like SLE and primary Sjogren’s syndrome. Its phase I trials are being prepared.

#### 3.2.3. Targeting IFN‐γ

AMG 811 is a monoclonal antibody that selectively blocks human IFN‐γ. In a Phase I single‐dose trial, AMG 811 dose dependently reduced serum CXCL10 levels and IFN‐γ pathway markers in lupus patients; however, it showed no significant efficacy in DLE treatment. AMG 811 was safe and well‐tolerated, with no anti‐drug antibodies detected [5, 116, 117]. Exploring combination therapies or optimized dosing could enhance its efficacy in DLE.

### 3.3. Targeting JAK‐STAT Pathway

Upon binding of IFN to its corresponding receptor, the JAK‐STAT pathway is activated. This pathway plays a crucial role in various inflammatory diseases, and significant progress has been made in treating these conditions with JAK inhibitors. In LE, several JAK inhibitors are under active development for LE to control symptoms and improve patients’ quality of life.

Deucravacitinib, a selective tyrosine protein kinase (TYK) 2 inhibitor, effectively inhibits TYK2 and STAT1 phosphorylation, as well as IFN‐*κ*, CXCL10, and IL‐6 expression in vitro [56]. In a Phase II study [92], oral Deucravacitinib 3 mg twice daily significantly improved CLASI50 response and skin symptoms among SLE patients at week 48 compared to placebo. However, AEs like rash and acne were more common in the Deucravacitinib group. It has also shown success in improving CLE, including recurrent SCLE [118, 119] and DLE [120]. Overall, Deucravacitinib demonstrates strong potential for treating SLE and CLE.

In a Phase II study, Upadacitinib (a JAK1 inhibitor) showed efficacy in treating SLE, both alone and in combination with Elsubrutinib (a Bruton’s Tyrosine Kinase inhibitor), reducing disease activity and improving CLASI50 response. These positive results have advanced Upadacitinib to a Phase III trial (NCT05843643), highlighting its potential as an SLE treatment [98].

Tofacitinib, a JAK1/3 inhibitor, has demonstrated promise in improving SLE disease activity, including nephritis, skin inflammation, and autoantibody production in mouse models [121]. It was well‐tolerated and safe in a Phase I study [122]. In a Chinese single‐center trial for SLE‐associated arthritis and rash [99], Tofacitinib rapidly improved arthritis symptoms, partially improved rash, and reduced glucocorticoid use without sAEs. However, among nine pediatric patients with refractory SLE, only four benefited clinically [123]. Further observation is needed to confirm its efficacy in SLE and CLE.

Ruxolitinib, a JAK1/2 inhibitor, significantly suppresses CXCL10 expression induced by nucleic acid analogs in human keratinocytes and CLE epidermal models in vitro [124]. It also markedly alleviates lupus‐like lesions in lupus‐prone mice [95]. In chilblain lupus patients, Ruxolitinib nearly resolves skin lesions [96, 97]. However, no clinical trial data are available for Ruxolitinib in treating LE.

## 4. Conclusions and Prospective Views

In CLE lesions, both stromal components and infiltrating immune cell populations exhibit upregulated ISG expression, collectively establishing a characteristic IFN signature. These cellular elements, along with IFN signaling, contribute to multifaceted pathological processes, such as immune cell recruitment, inflammatory responses, and fibrotic tissue remodeling. Emerging evidence from preclinical models and clinical trials indicates that therapeutic strategies targeting IFN pathways hold promise for disease intervention. Considering the suboptimal outcomes of current CLE management, IFN‐centric therapies may offer novel treatment paradigms through precisely modulated immunological targeting. Subsequent investigations should prioritize mechanistic elucidation of IFN axis involvement across distinct CLE subtypes, optimizing therapeutic protocols for enhanced clinical outcomes while systematically evaluating long‐term safety profiles.

**Nomenclature**
LE: Lupus erythematosus
CLE: Cutaneous lupus erythematosus
SLE: Systemic lupus erythematosus
SCLE: Subacute cutaneous lupus erythematosus
DLE: Discoid lupus erythematosus
UVR: Ultraviolet radiation
IFN: Interferon
ISG: IFN‐stimulated gene
PDC: Plasmacytoid dendritic cell
JAK‐STAT: Janus kinases‐signal transducer and activator of transcription
IFNAR: IFN‐α receptor
NK cell: Natural killer cell
IFNLR: IFN‐λ receptor
TLR: Toll‐like receptor
TGF‐β: Transforming growth factor‐β
ENA: Endogenous nucleic acid
CXCL: C─X─C motif ligand
CXCR: C─X─C motif receptor
IL: Interleukin
Th: T helper cell
Treg: Regulary T cell
Trm: Tissue‐resident memory T cell
BAFF: B cell activating factor
LC: Langerhans cell
ADAM17: A disintegrin and metalloproteinase 17
EGFR: Epidermal growth factor receptor
NET: Neutrophil extracellular trap
CLASI: Cutaneous Lupus Erythematosus Disease Area and Severity Index
BDCA: Blood dendritic cell antigen
ILT7: Immunoglobulin‐like transcript 7
AE: Adverse effect
SAE: Severe adverse effect
TYK: Tyrosine protein kinase
ZBP1: *Z*‐DNA‐binding protein 1
cGAS: Cyclic GMP–AMP synthase
STING: Stimulator of interferon genes
FoxP3: Forkhead box protein P3.

## Disclosure

All authors read and agreed to the published version of the manuscript.

## Conflicts of Interest

The authors declare no conflicts of interest.

## Author Contributions

**Min Gao:** conceptualization, writing – original draft. **Yumin Xia:** conceptualization, funding acquisition, writing – review and editing. **Nenghan Zhang:** review and editing.

## Funding

This study was funded by the National Natural Science Foundation of China (Grant 82473522), the Key Medical Research Plan of Xi’an City (Grant 24YXYJ0001), the Special Support Program for High‐Level Talents of Shaanxi Province, and the Innovation Capability Support Plan of Shaanxi Health Commission (Grant 2024TD‐15).

## Data Availability

Data sharing is not applicable to this article as no datasets were generated or analyzed during the current study.
